# Site-specific glycosaminoglycan content is better maintained in the pericellular matrix than the extracellular matrix in early post-traumatic osteoarthritis

**DOI:** 10.1371/journal.pone.0196203

**Published:** 2018-04-25

**Authors:** Simo P. Ojanen, Mikko A. J. Finnilä, Aino E. Reunamo, Ari P. Ronkainen, Santtu Mikkonen, Walter Herzog, Simo Saarakkala, Rami K. Korhonen

**Affiliations:** 1 Department of Applied Physics, University of Eastern Finland, Kuopio, Finland; 2 Research Unit of Medical Imaging, Physics and Technology, Faculty of Medicine, University of Oulu, Oulu, Finland; 3 Mechanical & Manufacturing Engineering, Schulich School of Engineering, University of Calgary, AB, Calgary, Canada; 4 Human performance laboratory, Faculty of Kinesiology, University of Calgary, AB, Calgary, Canada; 5 Department of Diagnostic Radiology, Oulu University Hospital, Oulu, Finland; University of South Carolina, UNITED STATES

## Abstract

**Introduction:**

One of the characteristics of early osteoarthritis (OA) is the loss of fixed charged density (FCD) of glycosaminoglycans in the superficial zone of articular cartilage. However, possible local changes in the FCD content of the pericellular matrix (PCM) are not fully understood. Hence, our aim was to investigate the effect of unilateral anterior cruciate ligament transection (ACLT) in rabbit knees on estimated FCD in the PCM compared to that in the ECM, and relate these results with cell morphology.

**Methods:**

Articular cartilage samples were collected from ACLT, contralateral and intact control knee joints from lateral and medial femoral condyles and tibial plateaus, and from the femoral groove and patella. Histological samples were prepared and stained with Safranin-O to estimate the FCD content around the chondrocytes in the PCM and the ECM with digital densitometry.

**Results:**

As a result of ACLT, the greatest decreases in the FCD content of the PCM were observed in the superficial zone of the lateral femoral condyle (*p* = 0.02), medial tibial plateau (*p* = 0.002) and patellar (*p* < 0.001) cartilage. The normalized FCD content of the PCM compared to the surrounding ECM was increased most in the femoral condyles (*p* < 0.01) and medial tibial plateau (*p* = 0.02) cartilage. The high normalized FCD content of the PCM in the superficial zone of lateral femoral condyle cartilage was consistent with the round cell morphology in that location.

**Conclusions:**

In conclusion, we suggest that certain sites in the knee joint, particularly the lateral femoral condyle cartilage, experience less FCD loss in the PCM than in the ECM in early post-traumatic OA, which could lead to altered cell shape.

## Introduction

Articular cartilage is mainly composed of chondrocytes and the extracellular matrix (ECM) which primarily consists of proteoglycans, collagen and interstitial fluid [1]. Proteoglycans are non-homogeneously distributed throughout the cartilage matrix and proteoglycan content increases with cartilage depth [2]. Proteoglycans contain a core protein to which negatively charged glycosaminoglycans are linked. Glycosaminoglycans lead to high fixed charge density (FCD) in articular cartilage, which contributes to the cartilage stiffness [3–6].

Chondrocytes’ main functions are to maintain the structure and composition of articular cartilage and produce ECM macromolecules, such as proteoglycans [7,8]. The shape of chondrocytes is highly dependent on the cartilage zone: in the superficial zone, chondrocytes are ellipsoid with their main axis parallel to the cartilage surface; in the middle zone, chondrocytes are round; and, in the deep zone, they are ellipsoidal with their main axis perpendicular to the cartilage surface [1,9]. Chondrocytes are surrounded by the pericellular matrix (PCM), which is mainly composed of proteoglycans, collagen and fluid [9,10]. The PCM’s main function is to protect chondrocyte during physiological loading by absorbing mechanical loads and preventing excessive stresses and strains [9].

Osteoarthritis (OA) is a severe and common knee joint disease in which, at the end stage, articular cartilage becomes eroded. Development and progression of post-traumatic OA can be studied using animal models of anterior cruciate ligament transection (ACLT) [11–19]. ACLT models have been shown to produce similar alterations in the physical properties of the articular cartilage as observed in humans during early OA [17].

One of the typical characteristics of early OA is the loss of proteoglycans and FCDs, especially in the superficial zone of articular cartilage [8,20]. Previous studies with ACLT rabbit models have shown that this degenerative change occurs in a highly site-specific manner in early OA [16,18]. Particularly, femoral condylar cartilages experienced severe proteoglycan loss in the ECM 4 weeks after ACLT. However, degenerative changes in the PCM composition have not been studied. On the other hand, it is known that proteoglycan synthesis of chondrocytes increases during early OA [21–24]. Therefore, one might expect less FCD loss in the PCM than in the ECM of rabbit cartilage as a result of ACLT.

Changes in cartilage composition during early OA alter the mechanical environment of chondrocytes [25]. These degenerative changes have been suggested to increase the aspect ratio (height/width) of superficial zone chondrocytes [26] and alter cell volume and shape in mechanically loaded cartilage [15,18]. Moreover, similar depth-wise alterations in the cell aspect ratio in osteoarthritic human hip joint cartilage was explained partly by proteoglycan loss in the ECM [27]. Particularly cells in the superficial zone became more round during OA. These alterations in chondrocyte morphology in OA cartilage are typically explained by the ECM structure and composition [27]. Due to the measurement scale, analysis of the ECM as a whole may result in a loss of identification in local tissue alterations that could be crucial to normal cell behavior. Therefore, a local analysis of the PCM is justified, with a focus on analyzing the FCD content, which has been suggested to alter cell volume more than other constituents in the PCM [28]. If there are alterations in the resting volume and shape or biomechanical behavior of cells due to alteration in the ECM or PCM properties, this might change the cartilage biosynthesis and accelerate the progression of OA [23,25].

Our aim was to investigate the effects of unilateral ACLT of rabbits on estimated FCD content in the PCM compared to that in the ECM 4 weeks after the ACLT, and relate these results with cell morphology. As proteoglycan synthesis in chondrocytes has been suggested to increase during early OA [21–24], we hypothesize that the relative FCD content in the PCM (normalized to that in the ECM) of rabbit cartilage is higher in the operated knees compared to the non-operated, control knees. That is, it is hypothesized that there is less FCD loss in the PCM than in the ECM. As the changes in the ECM proteoglycan content of articular cartilage are highly site-specific [16,18], we also hypothesize that the normalized FCD content in the PCM is site-specific. Moreover, as characterized previously, changes in the ECM structure are more ‘global’, thus, we hypothesize that an increase in the local normalized FCD content of the PCM occurs at sites that experience altered cell shape as a result of ACLT.

The greatest decreases in the FCD content of the PCM were observed in the superficial zone of the lateral femoral condyle, medial tibial plateau and patellar cartilage due to ACLT. The normalized FCD content of the PCM compared to the surrounding ECM was increased most in the femoral condyle and medial tibial plateau cartilage. Certain sites in the knee joint, particularly the lateral femoral condyle cartilage, experience less FCD loss in the PCM than in the ECM in early post-traumatic OA, which could lead to altered cell shape.

## Methods

### Animal model

**Fig 1:**
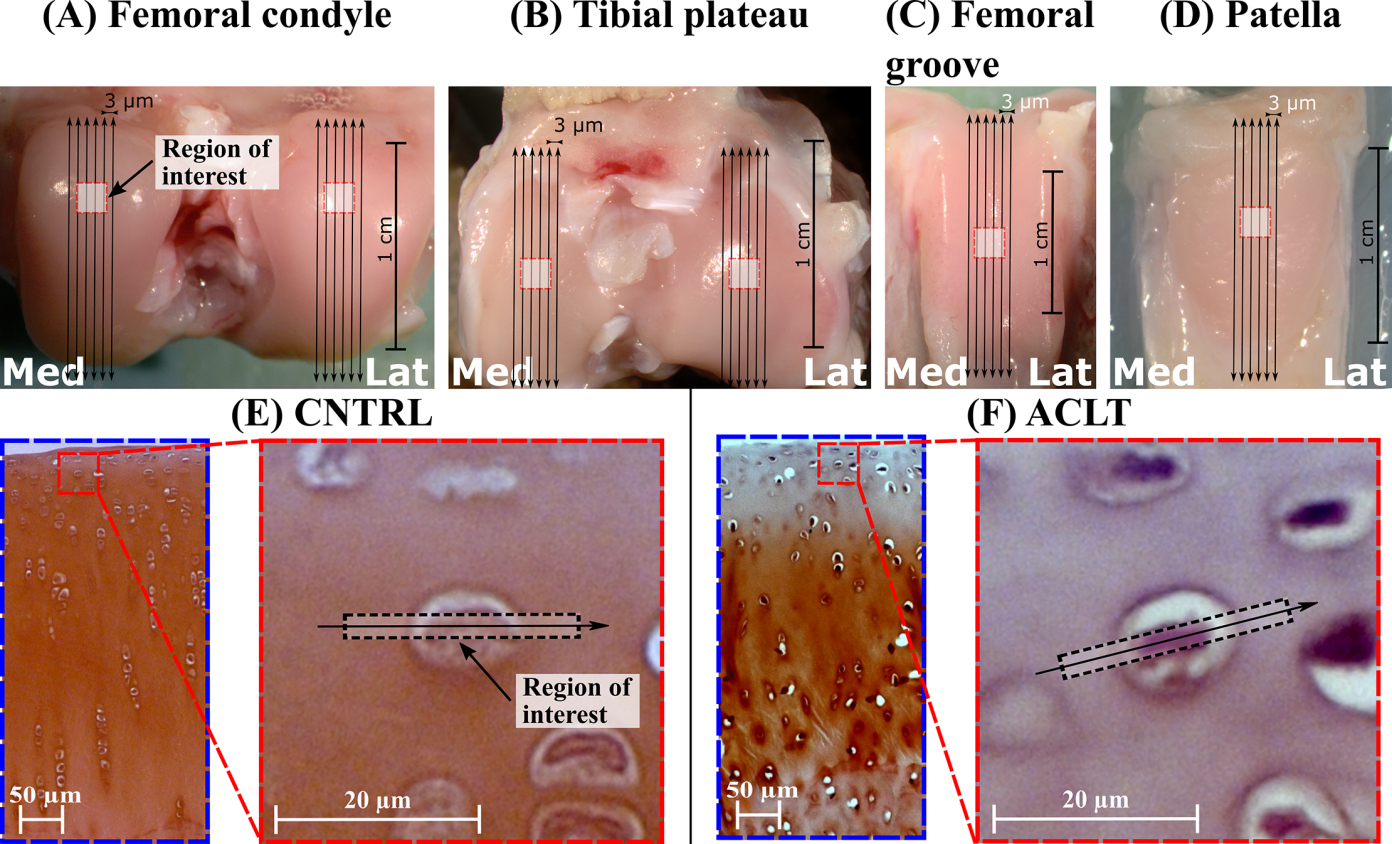
**Histological samples for digital densitometry analysis were prepared from both lateral and medial femoral condyles (A), tibial plateaus (B) and from the center of femoral groove (C) and patella (D). Sections were prepared perpendicular to the cartilage surface and parallel to the long axis of each sample site (illustrated as arrows). Region of interest from each section was collected from the primary load bearing area of each site: the highest point of the femoral condyles, the center of the tibial plateaus, the center of the femoral groove and patella. Representative images of the Safranin-O stained histological sections from medial femoral condyle cartilage of control (E) and operated (F) groups are also shown.** Lat, Lateral; Med, Medial; CNTRL, Control; ACLT, Anterior Cruciate Ligament Transection.

Samples were collected and prepared for the histology as described in previous studies [16,18]. All procedures were conducted according to the guidelines of the Canadian Council on Animal care and were approved by the Animal Ethics committee at the University of Calgary [16,18]. Briefly, nine skeletally mature female New Zealand white rabbits (Oryctolagus cuniculus, age 14 months, weight 5.4 ± 0.6 kg) had gone through unilateral ACLT surgery under general anesthesia. Anesthesia was induced by first delivering a pre-med sedative (SQ, Acepromazine maleate, 1 mg/kg body weight, AceVet®, Vétoquinol Inc., Lavaltrie, QC, Canada). 30 minutes later the animals were placed under deep surgical plane anesthesia using 5% Isoflurane (Fresenius Kabi Inc., Richmond Hill, ON, Canada) in medical oxygen (1 l/min). Surgical depth was maintained with 1 to 2% Isoflurane in medical oxygen. Animals were sacrificed 4 weeks after the surgery under general anesthesia and both operated and contralateral knee joints were harvested. In addition, six intact control knee joints from three rabbits were collected and used as a control group. Euthanasia was performed using an intravenous (ear vein) barbiturate overdose. Sodium pentobarbital (Euthanyl®, Bimeda-Mtc Animal Health Inc., Cambridge, ON, Canada) was infused at a dose of 200 mg/kg body weight. Lateral and medial femoral condyles and tibial plateaus, femoral groove and patella were removed for further sample processing (Fig 1). ACLT rabbit models have been shown to produce similar alterations in the physical properties of articular cartilage as observed in humans during early OA [17].

### Sample preparation

Samples were originally prepared for previous studies [16,18]. Briefly, samples were fixed in formalin, decalcified with ethylenediaminetetraacetic acid, dehydrated in a series of graded alcohols and embedded in paraffin [16,18]. Lateral and medial femoral condyles, tibial plateaus and the center of the femoral groove and patella were cut with a microtome (LKB 2218 HistoRange microtome, LKB produkter, Ab, Bromma, Sweden) along the mid axis, and 9 sections from the patella and 12–18 sections from the other locations were sectioned (thickness 3 μm) from each sample (Fig 1A–1D) and stained with Safranin-O (Fig 1E and 1F) [16,18]. Cationic Safranin-O binds stoichiometrically to the negatively charges (FCD) of proteoglycans [29,30], and digital densitometry can be used to estimate local FCD [31].

### Microscopic analysis

**Fig 2:**
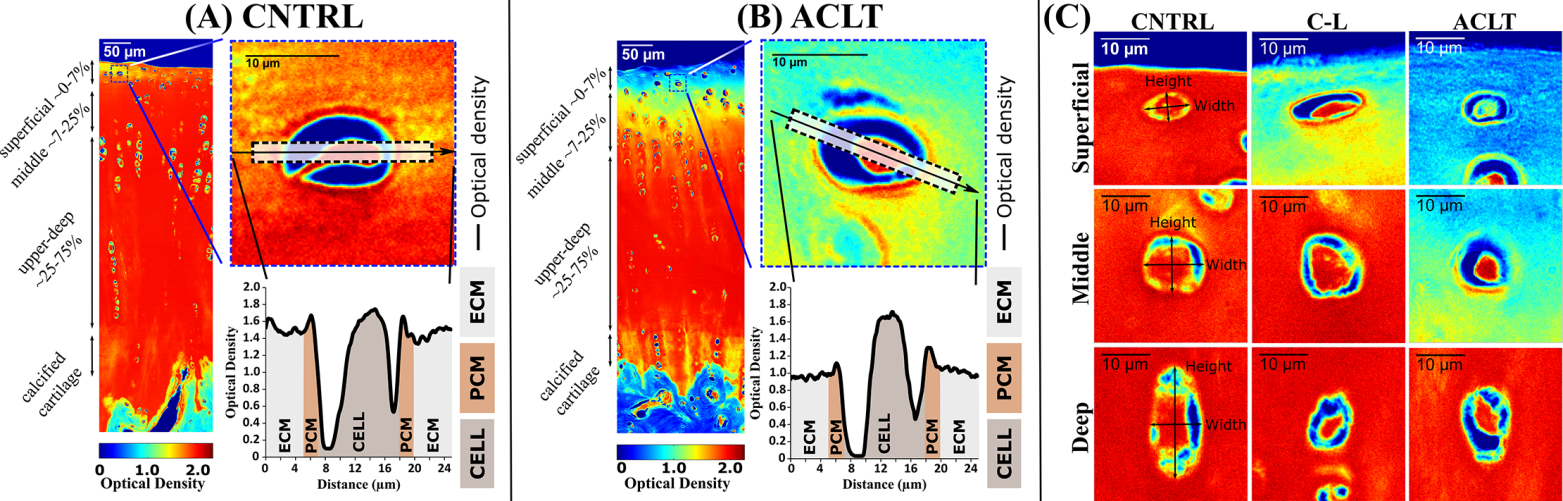
**Examples of optical density images acquired by digital densitometry imaging from medial femoral condyle cartilage tissue and analyzed optical density profiles in the horizontal axis around the chondrocytes in (A) control and (B) operated knees. Examples of optical density images around chondrocytes acquired by digital densitometry from control, contralateral and operated groups in the superficial, middle and deep zones of medial femoral condyle cartilage (C). The cell height and width were defined by the directions of the minor and major axis of the chondrocyte, respectively.** CNTRL, Control; C-L, Contralateral; ACLT, Anterior Cruciate Ligament Transection; PCM, Pericellular Matrix; ECM, Extracellular Matrix.

Digital densitometry imaging was conducted with the light microscope (Nikon Microphot FXA, Tokyo, Japan) equipped with a CCD cooled camera (Hamamatsu photonics K.K, Hamamatsu City, Japan, pixel size = 0.1563 μm, NA = 0.95). An image from each section was collected from the primary load bearing area of each site: the highest point of the femoral condyles, the center of the tibial plateaus, the center of the femoral groove and patella. These were the areas where biomechanical testing had been conducted in previous studies [16,18]. Imaging was performed with 40× magnification under monochromatic illumination (492 ± 5 nm). Digital densitometry images were calibrated against the neutral density filters (optical density values 0.0, 1.0, 1.3, 1.6, 2.0, 2.6 and 3.0) (Schott, Mainz, Germany). On average, 5 viable cells with their surroundings were analyzed from each section with a custom made MATLAB R2012a (Mathworks Inc., Natick, Massachusetts, United States) -script. Chondrocyte height, width and aspect ratio (height/width) were measured. The cells were analyzed from different and clearly defined zones (superficial: ~0–7%; middle: 7–25%; deep: 25–75% of the normalized cartilage thickness, down to the tidemark), with an average depth of 3.5% (95%CI 3.2–3.8%), 13.2% (95%CI 11.5–14.8%) and 36.8% (95%CI 34.0–39.5%) from the cartilage surface for the superficial, middle and deep zones (Fig 2A, 2B and 2C). Cells in the different zones were defined by their morphology as follows: in the superficial zone, chondrocytes were ellipsoid shaped and their main axis was parallel to the cartilage surface; in the middle zone, chondrocytes were round; and, in the deep zone, they were ellipsoid with their main axis perpendicular to the cartilage surface [1,9]. Cell height and width were defined by the directions of the minor and major axis of the chondrocyte respectively (Fig 2C). Cell width was approximately parallel to the cartilage surface, taking into account whether the cell was tilted (Fig 2C), and cell height was perpendicular to the width direction.

Optical density from digital densitometry was calculated as profiles in a direction parallel to the chondrocyte width (Fig 2A and 2B). First, a rectangular region of interest (height ~6 μm, width ~30 μm) was drawn over each chondrocyte and an optical density profile was generated by averaging image values perpendicular to the region width. Then, the maximum optical density values we assumed to represent the center of the PCM, and the final profile was generated by extending the analyzed area 40 pixels (6.25 μm) towards the ECM on both sides of the cell (Fig 2A and 2B). For visualization purposes, the PCM was defined to extend 1 μm towards the ECM and the cell center from the maximum optical density value (Fig 2A and 2B). Otherwise this information was not used in the analysis; either maximum values were used or point-by-point comparisons were conducted. The region of interest was limited to 6.25 μm towards the ECM to crop off nearby cells. In addition to the measured optical density (estimate of FCD), normalized optical density profiles were calculated in order to highlight the differences in the FCD content of the PCM compared to that of the ECM by normalizing optical density values to the first point of each profile, representing the FCD content in the ECM. The FCD content in the superficial zone of the articular cartilage changes radically within a few micrometers depth-wise towards the deeper zones [16,18] increasing uncertainties of the normalized FCD content around the chondrocytes (normalized to the ECM). Therefore, to minimize this uncertainty, the optical density profiles were analyzed only in a direction parallel to the articular cartilage surface where the ECM properties were relatively uniform within the analyzed region.

### Statistical analysis

Point-by-point statistical analysis was performed to the optical density profiles in order to compare the ACLT and contralateral groups to the control group in the superficial, middle and deep zones separately. Comparisons were made with a Linear Mixed Model [32] which takes into account the dependence between the samples and animals. In the model, each group as well as the distance from the ECM was set as a fixed-type variable, as animals in each group and samples from each animal were set as random-type variables. Statistical analysis was conducted using the measured and normalized optical density profiles as well as the cell widths, heights and aspect ratios. Bonferroni correction was used in multiple comparisons in order to get conservative estimates of significant differences between groups. Statistical analysis was made with SPSS Ver. 21 (IBM Corp., Armonk, NY)–software.

## Results

### Measured FCD content

**Fig 3:**
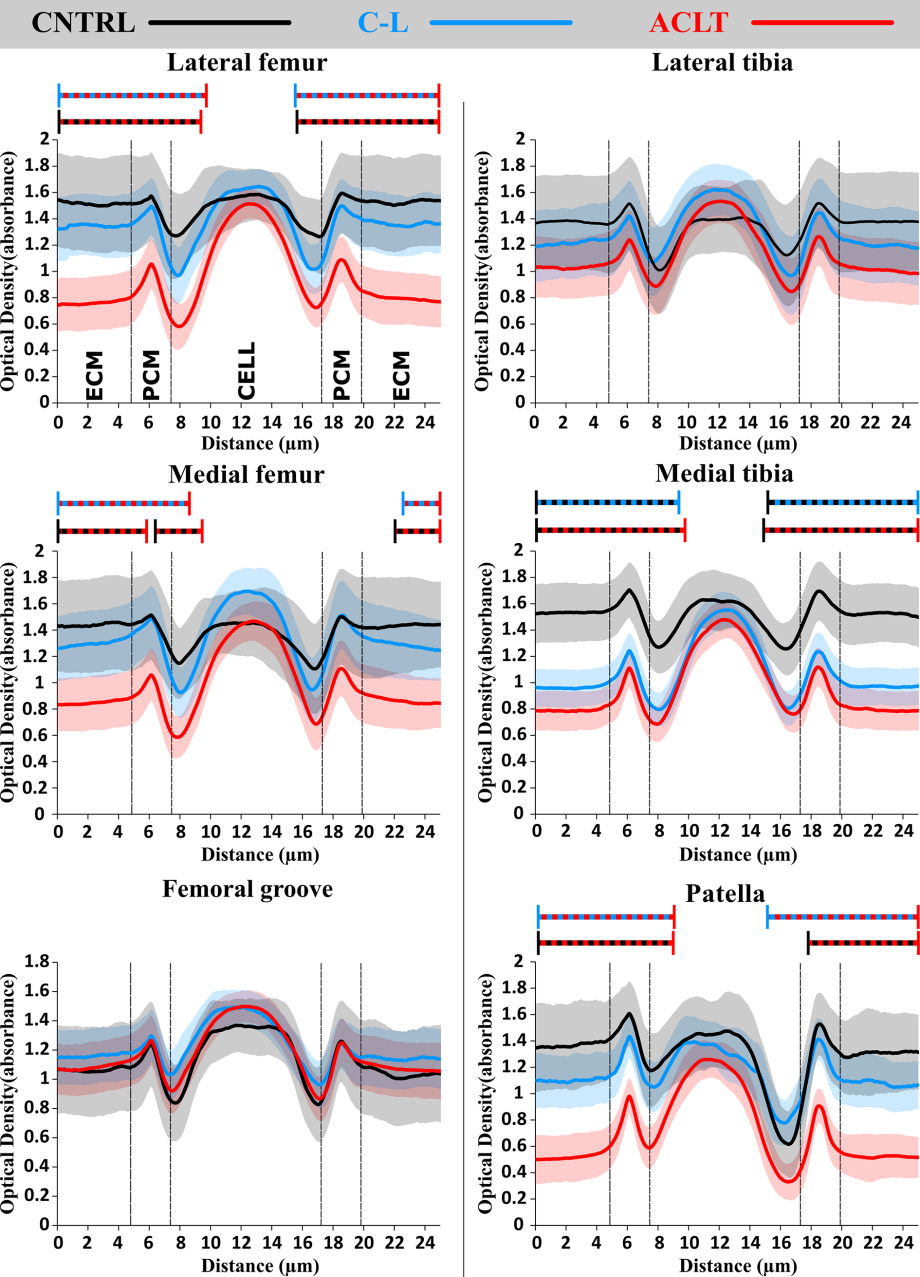
Measured optical density profiles in the vicinity of chondrocytes in the horizontal (transversal) direction from all analyzed locations in the superficial zone of cartilage: Lateral and medial femoral condyles and tibial plateaus and from the center of femoral groove and patella. **Red, blue and black lines represent means of operated, contralateral and control groups, respectively. Shaded areas around the colored lines represents the confidence intervals (95% CI) and the two colored dashed lines statistical difference (*p* < 0.05) between the color-coded groups**. ACLT, Anterior Cruciate Ligament Transection; C-L, Contralateral; CNTRL, Control; CI, Confidence Interval; PCM, Pericellular Matrix; ECM, Extracellular Matrix.

Measured FCD content of the PCM, as estimated from the optical density measurements, was significantly smaller in the ACLT group compared to the control group cartilage. Changes were observed in the superficial and middle zones of the lateral femoral condyle, the medial tibial plateau and the patellar cartilage (Fig 3, Table 1, S1 Fig), and they reached 6.25 μm (the edge of the analyzed area) from the center of the PCM towards the ECM. FCD loss of similar extent occurred also in the middle zone of the medial femoral condyle, and in the deep zone of the patellar cartilage (S1 Fig, S2 Fig).

**Table 1:** Significant differences (pointed by arrows) between the analyzed groups when comparing the measured and normalized optical density values at the highest point of the PCM.

***Measured***	*Groups*		
*Zone*	**ACLT vs CNTRL**	**ACLT vs C-L**	**C-L vs CNTRL**
*superficial*	↓ LatFC* / MedTP** / PAT**	↓ LatFC* / MedFC* /	↓ MedTP*
		MedTP* / PAT**	
*middle*	↓ LatFC* / MedFC** /	↓ LatFC** / MedFC* / PAT*	↓ MedTP*
	MedTP* / PAT**		
*deep*	↓ PAT*	↓ FG* / PAT**	–
***Normalized***	*Groups*		
*Zone*	**ACLT vs CNTRL**	**ACLT vs C-L**	**C-L vs CNTRL**
*superficial*	↑ LatFC** / MedFC** / MedTP*	↑ LatFC** / MedFC**	–
*middle*	↑ MedFC* / MedTP*	↑ LatFC* / MedFC*	–
*deep*	↑ PAT**	↑ PAT**	–

ACLT, Anterior Cruciate Ligament Transection; C-L, Contralateral; CNTRL, Control.

Lat, Lateral; Med, Medial; FC, Femoral Condyle; TP, Tibial Plateau; FG, Femoral Groove; PAT, Patella.

p-values were calculated using Bonferroni corrected pairwise comparison.

*p < 0.05

**p < 0.01

When comparing the ACLT to the contralateral group knees, significant FCD loss due to ACLT occurred in the superficial and middle zones of the lateral and medial femoral condyle, and in the patellar cartilage (Fig 3, Table 1, S1 Fig), where the differences reached the edge of the analyzed area. Furthermore, significant reductions in the FCD content of the PCM where observed in the deep zone of the femoral groove and patellar cartilage, and they reached the edge of the analyzed area as well (Table 1, S2 Fig). When comparing the contralateral to the control group knees, FCD content of the PCM was significantly decreased in the superficial and middle zones of the medial tibial plateau cartilage, and this decrease extended up to the edge of the analysis area (Fig 3, S1 Fig).

### Normalized FCD content

**Fig 4:**
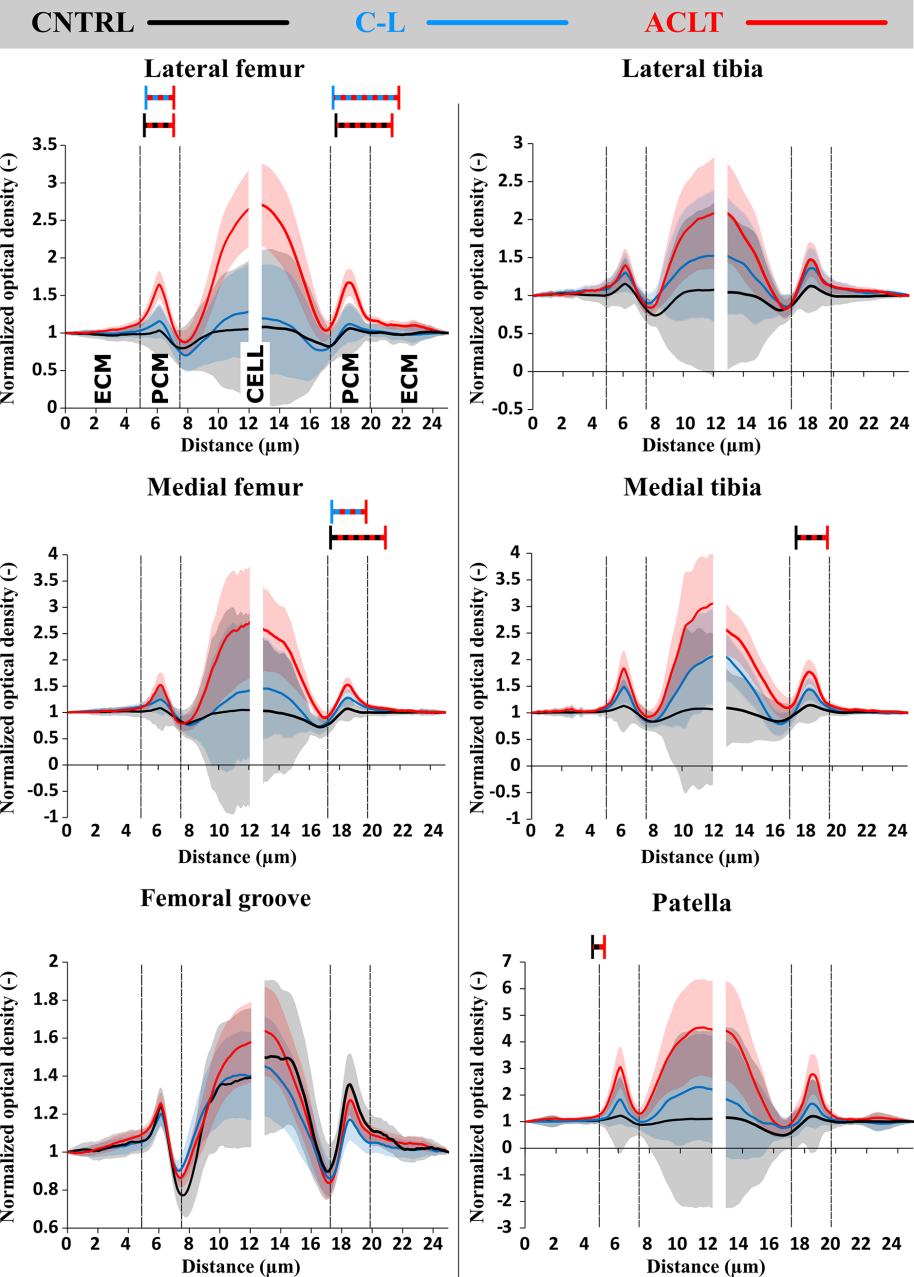
Normalized optical density profiles in the vicinity of chondrocytes in the horizontal (transversal) direction from all analyzed locations in the superficial zone of cartilage: Lateral and medial femoral condyles and tibial plateaus and from the center of femoral groove and patella. **Red, blue and black lines represent means of operated, contralateral and control groups, respectively. Shaded areas around the colored lines represents the confidence intervals (95% CI) and the two colored dashed lines statistical difference (*p* < 0.05) between the color-coded groups. Normalization was made to the both ends of the raw, un-normalized profiles and the optical density was analyzed from both sides of the cells separately.** ACLT, Anterior Cruciate Ligament Transection; C-L, Contralateral; CNTRL, Control; CI, Confidence Interval; PCM, Pericellular Matrix; ECM, Extracellular Matrix.

When comparing the ACLT to the control group knees, normalized FCD content of the PCM was generally increased in the ACLT group. Significant increases were observed in the superficial zone of the lateral and medial femoral condyle cartilage, and these increases extended 2.2 μm from the center of the PCM towards the ECM (Fig 4, Table 1). Increases in the normalized FCD content of the PCM were also present in the superficial zone of the medial tibial plateau cartilage, and they extended 1.1 μm from the center of the PCM towards the ECM (Fig 4, Table 1). Increased normalized FCD content of the PCM occurred in the middle zones of the medial femoral condyle and the medial tibial plateau cartilage, and this increased FCD content reached 1.6 μm and 0.6 μm from the center of the PCM towards the ECM, respectively (S3 Fig, Table 1). In the deep zone of the patellar cartilage, significant increases in the normalized FCD content of the PCM were observed and they reached 2.9 μm from the center of the PCM towards the ECM (S4 Fig, Table 1).

The normalized FCD content of the PCM was also increased in the ACLT group compared to the contralateral group knees. Changes were observed in the superficial and middle zones of the lateral and medial femoral condyle cartilage, and they reached 2.5 and 0.6 μm from the center of the PCM towards the ECM in the superficial zone, respectively, and 1.6 μm and 2.5 μm in the middle zone, respectively (Fig 4, Table 1, S3 Fig). In the deep zone of the patellar cartilage, increases in the normalized FCD content of the PCM reached 1.3 μm from the center of the PCM towards the ECM (S4 Fig, Table 1). Differences between the contralateral and control group knees were not significant.

### Cell shape

In ACLT group animals, cell height was significantly increased in the superficial zone of the lateral femoral condyle cartilage compared to the control group animals (Table 2, S1 Table). In the middle zone, cell height was significantly reduced in the lateral and medial femoral condyle, tibial plateau, and the patellar cartilages (Table 2, S2 Table). Cell height was also reduced in the middle zones of the lateral and medial tibial plateau cartilage from the contralateral to the control group and in the lateral femoral condyle cartilage from the ACLT to the contralateral group (Table 2, S2 Table).

**Table 2:** Significant differences (pointed by arrows) between the analyzed groups when comparing the cell height, width and aspect ratio (height/width).

*Cell height*	*Groups*			
*Zone*	**ACLT vs CNTRL**	**ACLT vs C-L**	**C-L vs CNTRL**	
*superficial*	↑ LatFC*	–	–	
*middle*	↓ LatFC* / MedFC* / LatTP** /	↓ LatFC**	↓ LatTP** / MedTP*	
* *	MedTP** / PAT**			
*deep*	–	–	–	
*Cell width*	*Groups*			
*Zone*	**ACLT vs CNTRL**	**ACLT vs C-L**	**C-L vs CNTRL**	
*superficial*	↓ LatFC** / MedTP*	↓ LatFC**	↓ MedTP**	
*middle*	↓ LatFC* / MedTP**	↓ MedFC**	↓ MedTP**	
*deep*	↓ FG*	↑ MedTP*	↓ FG** / MedTP**	
* *				
*Cell aspect ratio*	*Groups*			
*Zone*	**ACLT vs CNTRL**	**ACLT vs C-L**	**C-L vs CNTRL**	
*superficial*	↑ LatFC**	↑ LatFC**	–	
*middle*	–	–	–	
*deep*	–	–	–	

ACLT, Anterior Cruciate Ligament Transection; C-L, Contralateral; CNTRL, Control.

Lat, Lateral; Med, Medial; FC, Femoral Condyle; TP, Tibial Plateau; FG, Femoral Groove; PAT, Patella.

p-values were calculated using Bonferroni corrected pairwise comparison.

*p < 0.05

**p < 0.01

Cell width was significantly decreased in the superficial zone of the lateral femoral condyle and medial tibial plateau cartilage in the ACLT group compared to the control group, and in the medial tibial plateau cartilage in the contralateral group compared to the control group (Table 2, S1 Table). In addition, difference between the ACLT and contralateral group occurred in the lateral femoral condyle cartilage (Table 2, S1 Table). In the middle zone, a significant decrease in cell width due to ACLT (compared to the control group) was also observed in the lateral femoral condyle and medial tibial plateau cartilages (Table 2, S2 Table). Similar decrease was observed in the medial tibial plateau cartilage from the contralateral to the control group and in the lateral femoral condyle cartilage from the contralateral to the ACLT group (Table 2, S2 Table). In the deep zone, a significant reduction in the cell width was observed in the femoral groove cartilage in the ACLT and contralateral groups compared to the control group rabbits (Table 2, S3 Table). In this zone of the medial tibial plateau cartilage, a significant decrease in the cell width was also observed from the contralateral to the control group, and an increase from the ACLT to the contralateral group (Table 2, S3 Table).

Significant differences in the cell aspect ratio (height divided by width) were only observed in the superficial zone of the lateral femoral condyle cartilage (Table 2, S1 Table). At this location, the cell aspect ratio was increased in the ACLT group cartilage compared to the control and contralateral group cartilage, indicating more round cells as a result of ACLT. We also found a negative correlation (*r* = -0.484, *p <* 0.001) between the maximum measured FCD in the PCM and cell aspect ratio and a positive correlation (*r* = 0.338, *p <* 0.001) between the maximum normalized FCD content in the PCM and cell aspect ratio (S5 Fig).

## Discussion

**Fig 5:**
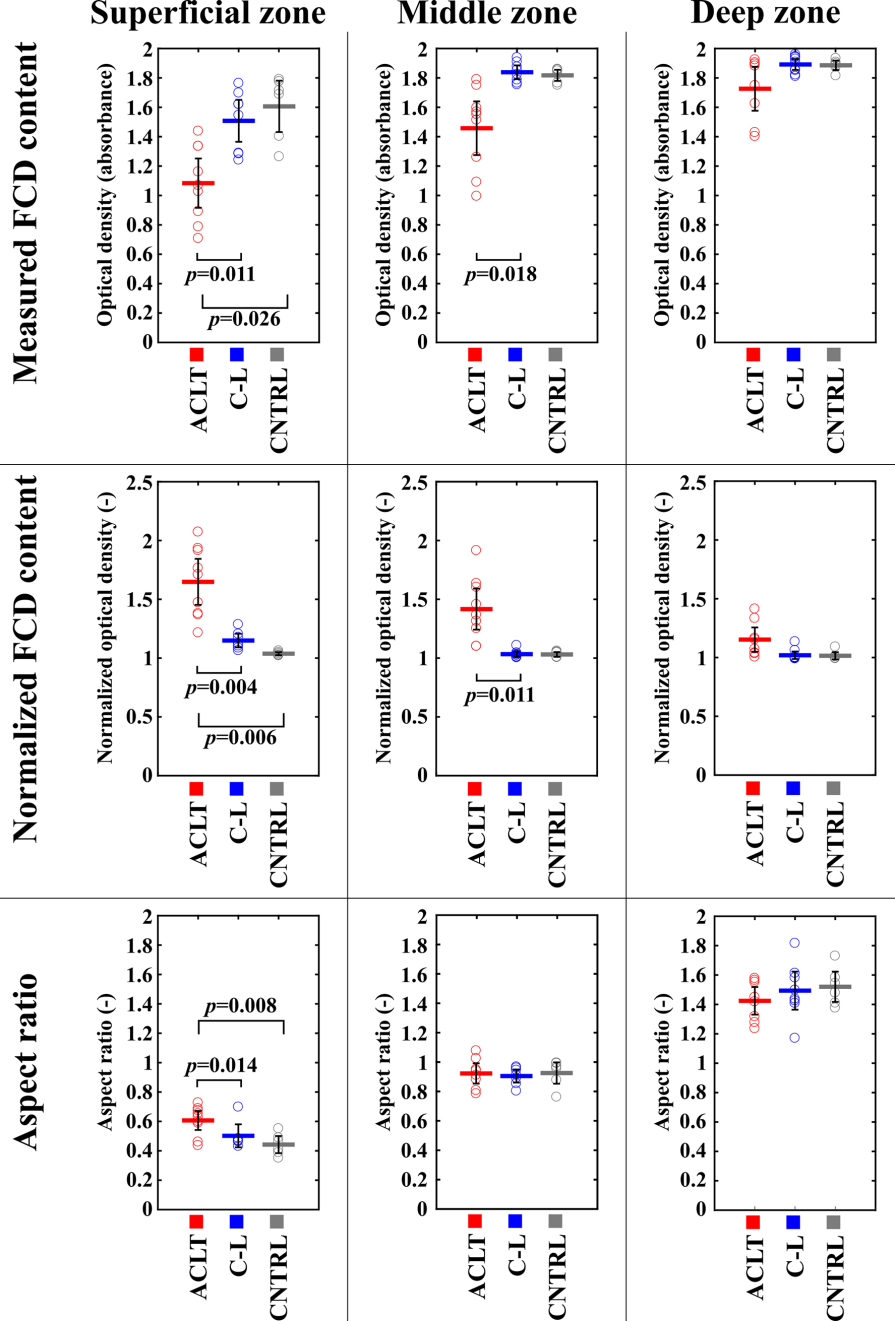
Measured and normalized fixed charged density content in the center of the pericellular matrix (the maximum value of the optical density profile) and cell aspect ratio in the superficial, middle and deep zones of the lateral femoral condyle cartilage with 95% confidence intervals. **Red, blue and gray colors represents the values of operated, contralateral and control groups, respectively. *p*-values were calculated using Bonferroni corrected pairwise comparison.** ACLT, Anterior Cruciate Ligament Transection; C-L, Contralateral; CNTRL, Control.

**Fig 6:**
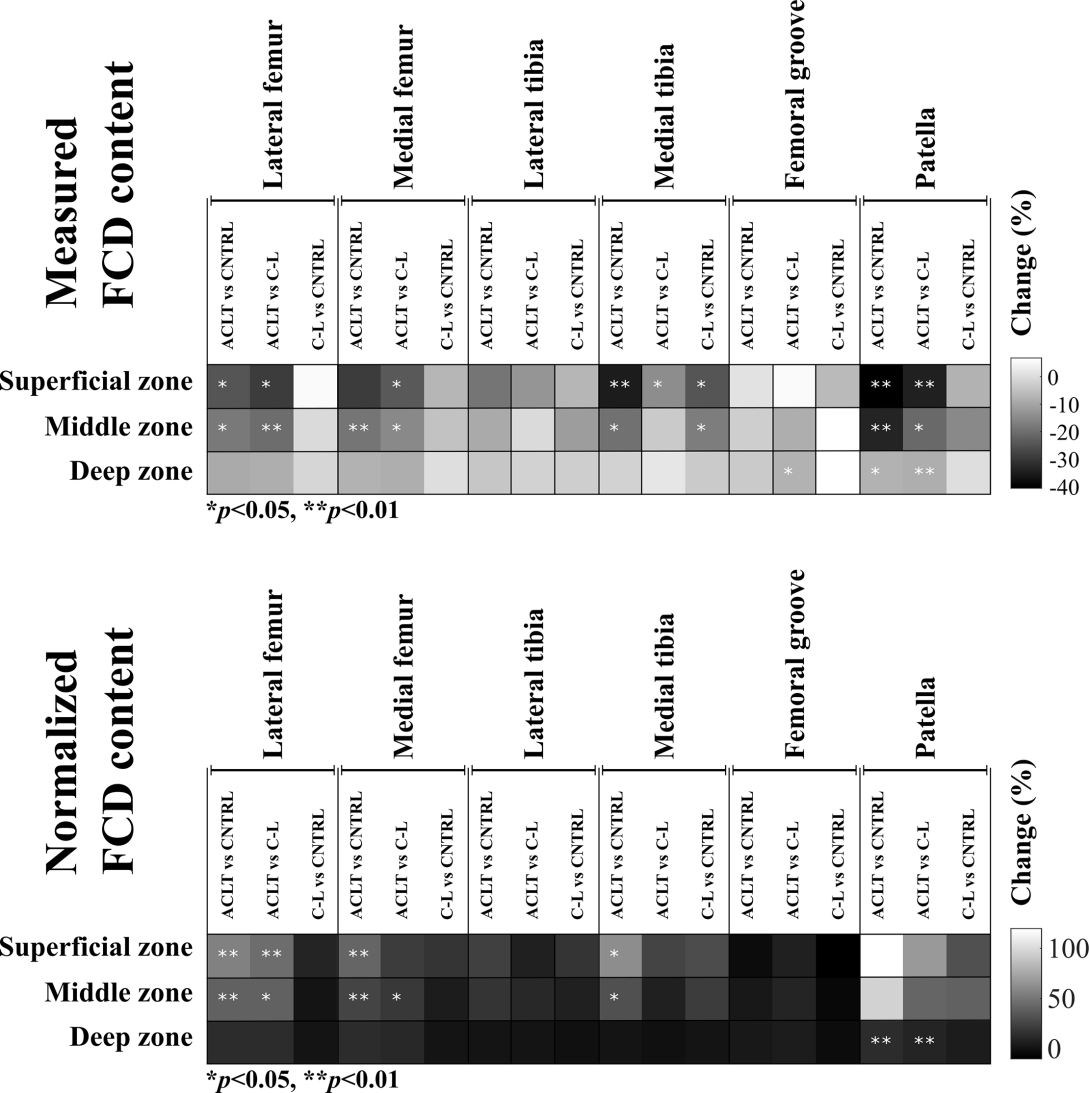
Summary of the changes in the measured and normalized fixed charged density contents in the center of the pericellular matrix (the maximum value of the optical density profile) in all analyzed sites and cartilage zones. ***p*-values were calculated using Bonferroni corrected pairwise comparison.** ACLT, Anterior Cruciate Ligament Transection; C-L, Contralateral; CNTRL, Control; FCD, Fixed Charged Density. *p<0.05 **p<0.01.

In this study, alterations in the estimated FCD content between the PCM and the ECM were investigated in an early stage of post-traumatic OA in an ACLT rabbit model. This analysis was supplemented by an evaluation of cell shapes at different tissue depths. Our results suggested that, as a result of ACLT, the FCD content in the PCM decreased in a site-specific manner, and this FCD loss was greatest in the superficial zone, consistent with previously observed alterations at the full tissue level [16,18] (Figs 5 and 6, Tables 1 and 2). The greatest increases in normalized FCD content due to ACLT were found in the lateral and medial femoral condyle and in the medial tibial plateau cartilage (Fig 6). Particularly high normalized FCD contents in the PCM of the lateral femoral condyle cartilage were consistent with more round cells in the superficial zone at this location (Fig 5).

The amount of FCDs was decreased in the PCM in the superficial and middle zones of the lateral and medial femoral condyle cartilage, medial tibial plateau cartilage and patellar cartilage. Alterations in the FCD content were consistent with the previous analysis on the full tissue level, where significant proteoglycan loss was observed at the same locations as observed here using local analysis procedures [16,18]. Furthermore, as in previous studies [16,18], proteoglycan loss was highly site-specific. Mäkelä et. al. [16] suggested that the first changes in the articular cartilage structure and composition as a result of ACLT occur primarily in the superficial zone of femoral condyle cartilages. This result is in line with the current PCM level analysis. However, the ECM in the femoral groove and lateral tibial plateau cartilage also experienced early alterations in proteoglycan content [16,18], while we did not observe changes in the FCD content in the PCM. It might be that FCDs remained at these locations in the PCM and territorial matrix (our analyzed region), while the inter-territorial matrix experienced these alterations.

An increased cell aspect ratio was observed only in the superficial zone of the lateral femoral condyle cartilage, indicated by more round cells in this area due to ACLT. At the same location, FCD content was reduced in the ECM and PCM, and the normalized FCD content was increased in the PCM compared to that in the ECM. A negative correlation was also observed between the measured FCD in the PCM and cell aspect ratio and a positive correlation between the normalized FCD in the PCM and cell aspect ratio. This result is consistent with an earlier numerical model showing that a decreased proteoglycan content in the PCM and ECM increased the cell aspect ratio [33]. This model also predicted that a decrease in proteoglycan content in the ECM without any change in the PCM (leading to increased normalized proteoglycan content in the PCM) leads to increases in the cell aspect ratio in the superficial zone of cartilage. Moreover, another numerical model of advanced OA cartilage showed that a decreased proteoglycan content in the ECM and PCM increases the cell aspect ratio [26].

In addition to the changes in cell shape, cell width was reduced in the superficial and middle zones of the lateral femoral condyle cartilage. This occurred also in the medial tibial plateau cartilage. Consistently, these locations experienced most alterations in measured and normalized FCD content in and around the PCM. Based on our earlier study of these same samples, alterations in the ECM collagen orientation were observed at these two locations [16], which likely also affects cell morphology.

In our study, differences between the lateral and medial knee compartments were observed. For instance, the PCM in the medial tibial plateau cartilage showed more alterations in measured and relative FCD content compared to those observed in the lateral tibial plateau cartilage. However, earlier results obtained from the ECM showed slightly more changes in the superficial zone of the lateral tibial plateau cartilage [16], while increased Mankin scores were only reported for the medial tibial plateau. It might be that morphological changes of the cells and their local FCD environment, as observed in the current study, contributed significantly to the Mankin score analysis [34].

Normally, higher contact forces are observed in the lateral, compared to the medial, compartment of the rabbit tibiofemoral joint during hopping [35]. ACLT causes instability of the knee joint, which changes rotations and translations of the tibia relative to the femur, possibly causing further alterations in the weight bearing sites, increasing the load on the lateral side of the knee joint. This scenario could explain the pronounced changes in the lateral femoral condyle cartilage observed in the present study. Thinning of the subchondral bone plate, decreased trabecular volume fraction, and decreased thickness have been observed only in the medial compartment of the femoral condyles, which might suggest a decreased stress to the medial compartment cartilage, and therefore less cartilage degradation, while the lateral side does not appear to have this “protective” mechanism [36]. On the other hand, in a lapine ACLT model, severe changes in articular cartilage have also been found in the medial knee compartment [19,37]. These results could partly explain our observed changes in the medial tibial cartilage, but not those seen in the lateral compartment. However, it should be noted that typical OA features, such as cartilage erosion, have been reported at knee locations other than the medial compartment as well [16,18,19,37].

As a result of ACLT, patellar cartilage had a significantly decreased measured FCD content in the PCM in all zones, while adjacent femoral groove cartilage did not show any changes. On the tissue level, changes in proteoglycan content between the femoral groove and patellar cartilage were similar as the amount of proteoglycans was decreased in the top ~20% in the femoral groove and top ~24% in the patellar cartilage [16,18]. Differences in the cell level FCD content might be caused by the way the boundaries of the regions of interest were chosen (6.25 μm from the center of the PCM towards the ECM), as cell level changes in the patellar cartilage occurred within this region, but changes in the femoral groove cartilage occurred further away from the cells (Figs 3 and 4, S1–S4 Figs). It might also be that in early OA, the ECM is more vulnerable to FCD loss than the PCM. Moreover, differences between ECM and PCM could be also due to the fact that the site analyzed from the femoral groove is only occasionally in contact with the patella, while the site from the patella is in contact with some part of the femoral groove at all times [16,38].

Differences in measured FCD content were also present between the contralateral and control groups. This might be caused by the asymmetrical loading between the surgical and intact contralateral knees, as it is known that the operated knee affects the mechanics in the contralateral knee [39]. However, the normalized FCD contents were similar between the contralateral and control groups, indicating simultaneous FCD loss in the PCM and ECM.

It is challenging to determine if chondrocytes were cut exactly in the center for the histological analysis. Off-center cutting may result in variations in cell width and height. In order to minimize this error, all samples and cells were prepared and analyzed in the same manner and the number of cells used for analysis was maximized. Furthermore, small confidence intervals suggest that the cells were consistently analyzed (S1–S3 Tables). The cell dimensions measured in this study were also in agreement with previous studies where chondrocyte dimensions were obtained from 3D confocal microscopy images of living cells [15,18,40]. In the superficial zone of the patellar cartilage, cell height and width were 8.3 μm and 11.1 μm, respectively, in the ACLT group, and 9.2 μm and 11.0 μm in the contralateral group. Cell height and width obtained using confocal imaging of the same samples were 5.7 μm and 12.6 μm respectively, in the ACLT group and 6.5 μm and 14.0 μm in the contralateral group [18]. In the control group, cell height and width were 9.1 μm and 12.0 μm, respectively, in the superficial zone of the patellar cartilage. The corresponding values from confocal imaging of age-matched (but not the same) samples were 5.0 μm and 11.3 μm for the cell height and width, respectively [40]. Small differences in cell height might result from slightly different analysis depth from the cartilage surface. Cells analyzed with the confocal microscope were closer to the cartilage surface and therefore slightly flatter.

Early OA, induced by ACLT, was associated with less FCD loss in the PCM than in the ECM, especially in the superficial and middle zones of the cartilage. There are at least two explanations for this finding: (i) either FCDs are bound more tightly in the PCM than in the ECM or (ii) FCD production in the chondrocytes is increased in early OA; or combination of these two possibilities. Changes in knee joint loading alters the metabolic activity of chondrocytes. ACLT in the canine knee joint has been associated with increases in proteoglycan turnover and production; supporting explanation (ii) [8,21,22,41]. Numerical simulation provided evidence that a decrease in the FCD associated with ACLT had a significant effect on cell volume during cartilage loading [28], which might lead to a further increase in FCD synthesis. FCD content in the PCM may also affect the biomechanical properties of the PCM, thereby altering the stresses and strains experienced by chondrocytes, possibly triggering FCD turnover in chondrocytes. Nonetheless, independent of which mechanism caused the observed results, FCD content in the PCM with respect to that in the ECM might have caused changes in cell shape, which might alter cartilage mechanotransduction. Gene expression analysis of various core proteins might give more insight into these questions.

In conclusion, we suggest that at certain sites in the knee joint, particularly in the lateral femoral condyle cartilage, FCD loss in the PCM is relatively low compared to the ECM in early post-traumatic OA in this rabbit model of ACLT. This finding may help explain the altered cell shape at this site in early OA. Thus, we provide novel information about PCM composition and structural changes in early post-traumatic OA, and how these changes might modulate the chondrocyte shapes. By modulating the FCD content in the PCM with respect to that in the ECM might potentially be used when targeting treatments for osteoarthritis, especially after trauma.

## Supporting information

S1 Fig Measured optical density profiles in the vicinity of chondrocytes in the horizontal (transversal) direction from all analyzed locations in the middle zone of cartilage: Lateral and medial femoral condyles and tibial plateaus and from the center of femoral groove and patella. **Red, blue and black lines represent means of operated, contralateral and control groups, respectively. Shaded areas around the colored lines represents the confidence intervals (95% CI) and the two colored dashed lines statistical difference (*p* < 0.05) between the color coded-groups.** ACLT, Anterior Cruciate Ligament Transection; C-L, Contralateral; CNTRL, Control.
(TIF)

S2 Fig Measured optical density profiles in the vicinity of chondrocytes in the horizontal (transversal) direction from all analyzed locations in the deep zone of cartilage: Lateral and medial femoral condyles and tibial plateaus and from the center of femoral groove and patella. **Red, blue and black lines represent means of operated, contralateral and control groups, respectively. Shaded areas around the colored lines represents the confidence intervals (95% CI) and the two colored dashed lines statistical difference (*p* < 0.05) between the color-coded groups.** ACLT, Anterior Cruciate Ligament Transection; C-L, Contralateral; CNTRL, Control.
(TIF)

S3 Fig Normalized optical density profiles in the vicinity of chondrocytes in the horizontal (transversal) direction from all analyzed locations in the middle zone of cartilage: Lateral and medial femoral condyles and tibial plateaus and from the center of femoral groove and patella. **Red, blue and black lines represent means of operated, contralateral and control groups, respectively. Shaded areas around the colored lines represents the confidence intervals (95% CI) and the two colored dashed lines statistical difference (*p* < 0.05) between the color-coded groups. Normalization was made to the both ends of the raw, un-normalized profiles and the optical density was analyzed from both sides of the cells separately.** ACLT, Anterior Cruciate Ligament Transection; C-L, Contralateral; CNTRL, Control.
(TIF)

S4 Fig Normalized optical density profiles in the vicinity of chondrocytes in the horizontal (transversal) direction from all analyzed locations in the deep zone of cartilage: Lateral and medial femoral condyles and tibial plateaus and from the center of femoral groove and patella. **Red, blue and black lines represent means of operated, contralateral and control groups, respectively. Shaded areas around the colored lines represents the confidence intervals (95% CI) and the two colored dashed lines statistical difference (*p* < 0.05) between the color-coded groups. Normalization was made to the both ends of the raw, un-normalized profiles and the optical density was analyzed from both sides of the cells separately.** ACLT, Anterior Cruciate Ligament Transection; C-L, Contralateral; CNTRL, Control.
(TIF)

S5 Fig **Correlation plot of the measured optical density and cell aspect ratio (*r* = -0.484, *p* < 0.001, R**^**2**^ **= 0.23) (A) and normalized optical density and cell aspect ratio (*r* = 0.338, *p* < 0.001, R**^**2**^ **= 0.12) (B) in the lateral femoral condyle cartilage. Red, blue and black dots represents the data of operated, contralateral and control groups, respectively.** ACLT, Anterior Cruciate Ligament Transection; C-L, Contralateral; CNTRL, Control.
(TIF)

S1 Table Mean values (95% CI) of the cell height, width and aspect ratio (height divided by width) in the superficial zone of the femoral groove, patella and lateral and medial femoral condyle and tibial plateau. * *p*<0.05, compared to the control group, ** *p*<0.05, comparison between the operated and contralateral groups. ACLT, Anterior Cruciate Ligament Transection; C-L, Contralateral; CNTRL, Control.
(DOCX)

S2 Table Mean values (95% CI) of the cell height, width and aspect ratio (height divided by width) in the middle zone of the femoral groove, patella and lateral and medial femoral condyle and tibial plateau. * *p*<0.05, compared to the control group, ** *p*<0.05, comparison between the operated and contralateral groups. ACLT, Anterior Cruciate Ligament Transection; C-L, Contralateral; CNTRL, Control.
(DOCX)

S3 Table Mean values (95% CI) of the cell height, width and aspect ratio (height divided by width) in the deep zone of the femoral groove, patella and lateral and medial femoral condyle and tibial plateau. * *p*<0.05, compared to the control group, ** *p*<0.05, comparison between the operated and contralateral groups. ACLT, Anterior Cruciate Ligament Transection; C-L, Contralateral; CNTRL, Control.
(DOCX)

S1 File Site-specific optical density profiles around the chondrocytes from the superficial, middle and deep zone cartilage. (ZIP)
