# Supplementary figures and images for: Site-specific glycosaminoglycan content is better maintained in the pericellular matrix than the extracellular matrix in early post-traumatic osteoarthritis
Source: PLoS One. 2018 Apr 25;13(4):e0196203. doi: 10.1371/journal.pone.0196203 (PMC5919041; doi:10.1371/journal.pone.0196203)

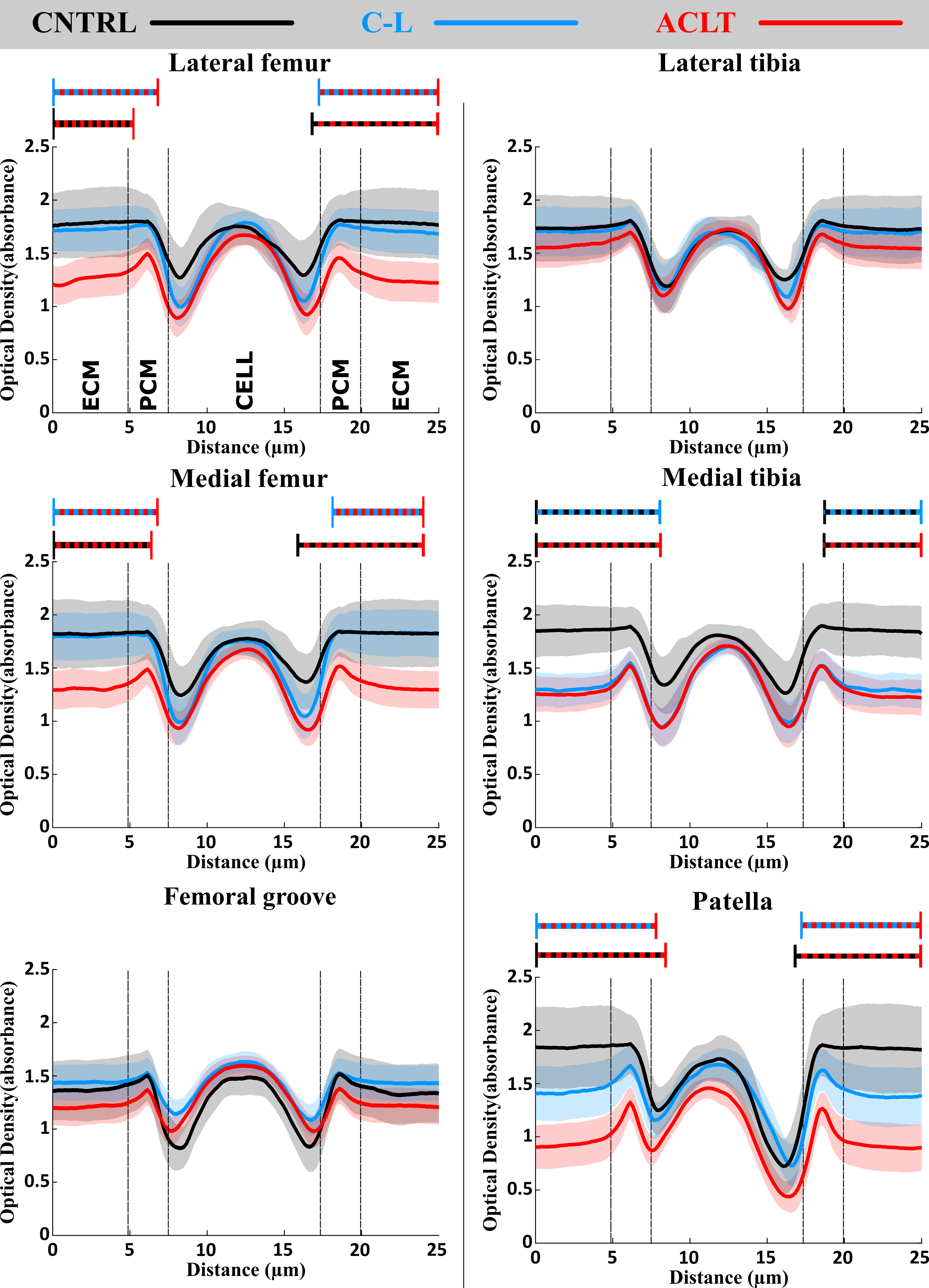

Supplement: S1 Fig — Red, blue and black lines represent means of operated, contralateral and control groups, respectively. Shaded areas around the colored lines represents the confidence intervals (95% CI) and the two colored dashed lines statistical difference (p < 0.05) between the color coded-groups. ACLT, Anterior Cruciate Ligament Transection; C-L, Contralateral; CNTRL, Control. (TIF) [file pone.0196203.s001.tif]

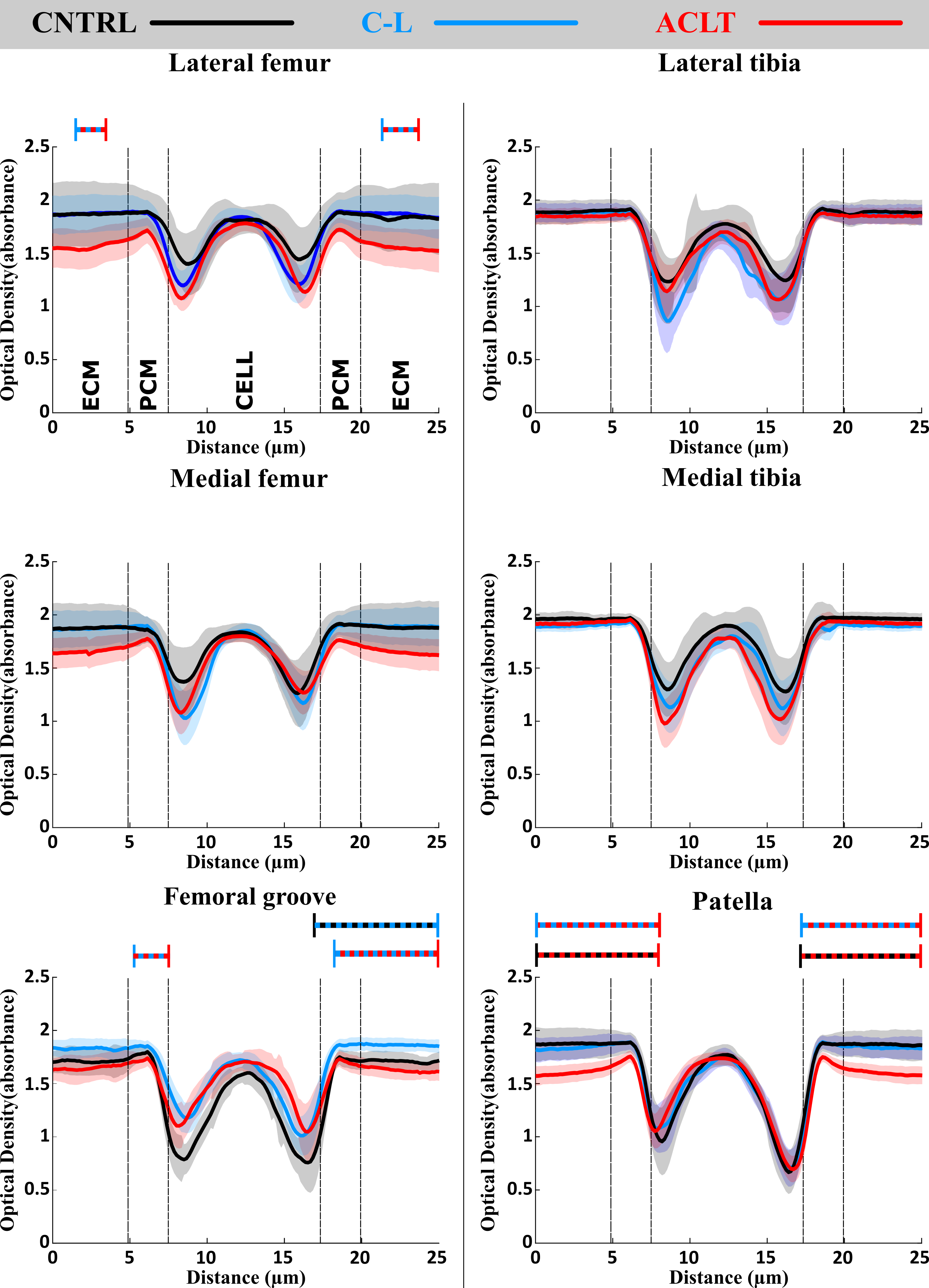

Supplement: S2 Fig — Red, blue and black lines represent means of operated, contralateral and control groups, respectively. Shaded areas around the colored lines represents the confidence intervals (95% CI) and the two colored dashed lines statistical difference (p < 0.05) between the color-coded groups. ACLT, Anterior Cruciate Ligament Transection; C-L, Contralateral; CNTRL, Control. (TIF) [file pone.0196203.s002.tif]

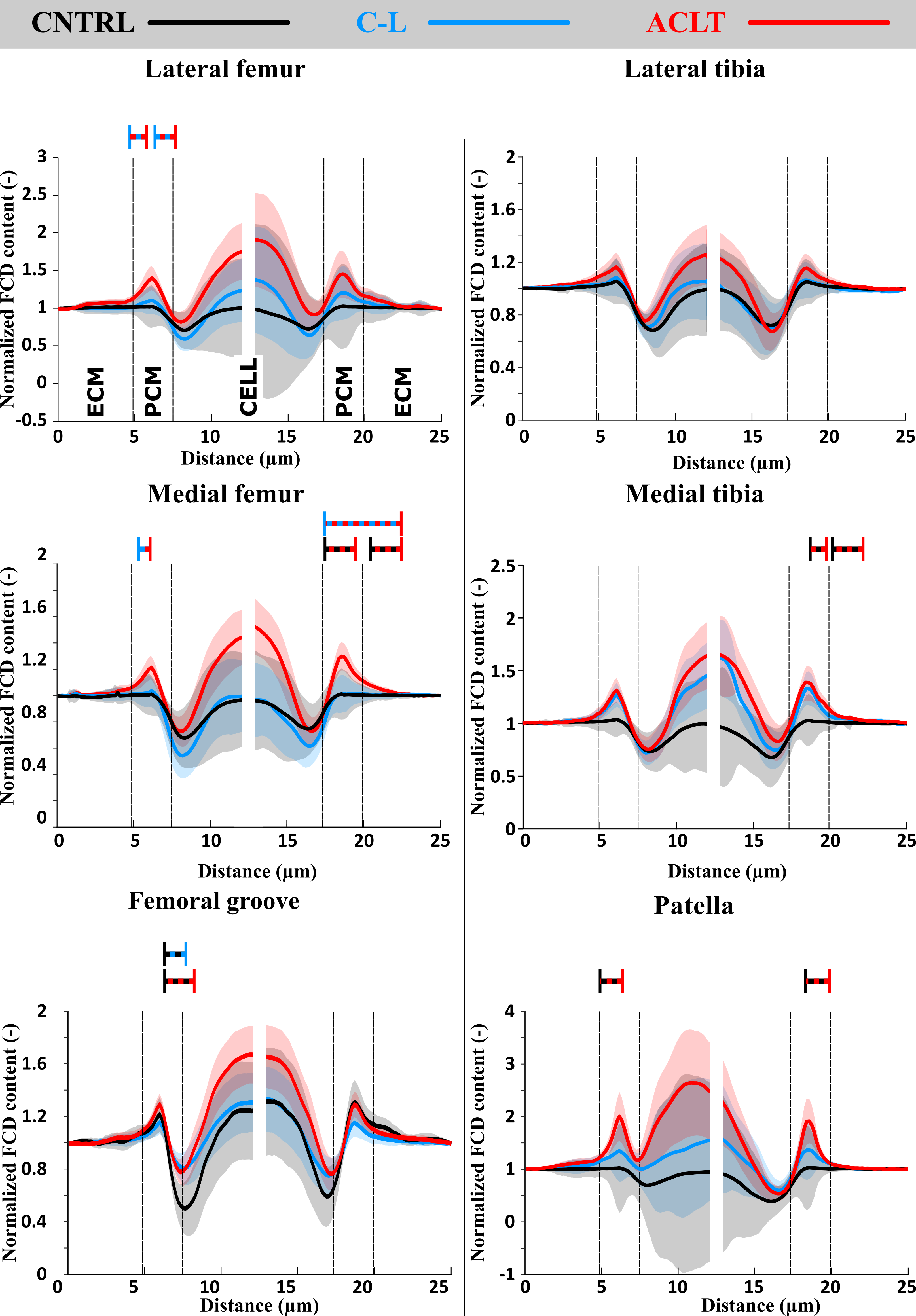

Supplement: S3 Fig — Red, blue and black lines represent means of operated, contralateral and control groups, respectively. Shaded areas around the colored lines represents the confidence intervals (95% CI) and the two colored dashed lines statistical difference (p < 0.05) between the color-coded groups. Normalization was made to the both ends of the raw, un-normalized profiles and the optical density was analyzed from both sides of the cells separately. ACLT, Anterior Cruciate Ligament Transection; C-L, Contralateral; CNTRL, Control. (TIF) [file pone.0196203.s003.tif]

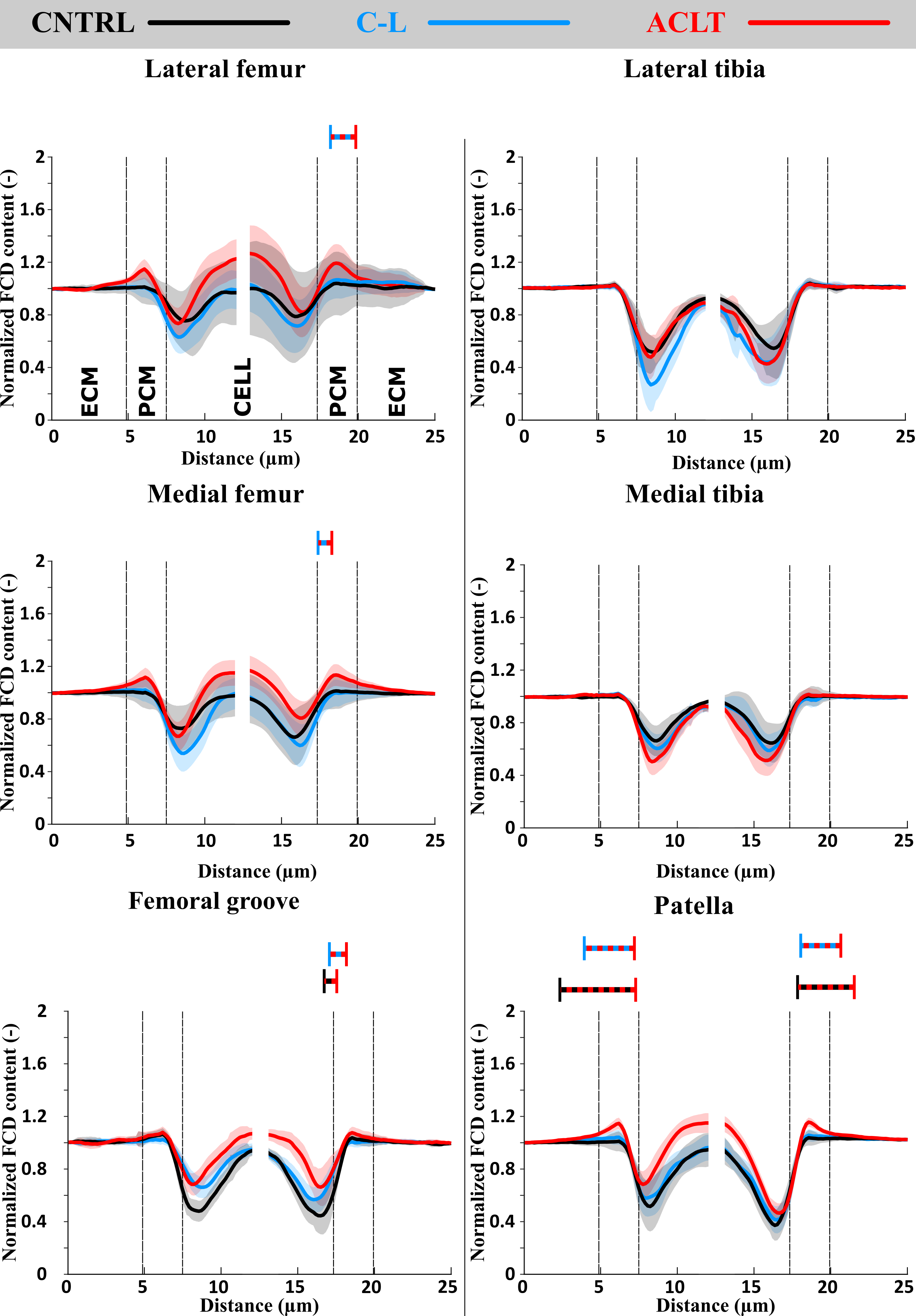

Supplement: S4 Fig — Red, blue and black lines represent means of operated, contralateral and control groups, respectively. Shaded areas around the colored lines represents the confidence intervals (95% CI) and the two colored dashed lines statistical difference (p < 0.05) between the color-coded groups. Normalization was made to the both ends of the raw, un-normalized profiles and the optical density was analyzed from both sides of the cells separately. ACLT, Anterior Cruciate Ligament Transection; C-L, Contralateral; CNTRL, Control. (TIF) [file pone.0196203.s004.tif]

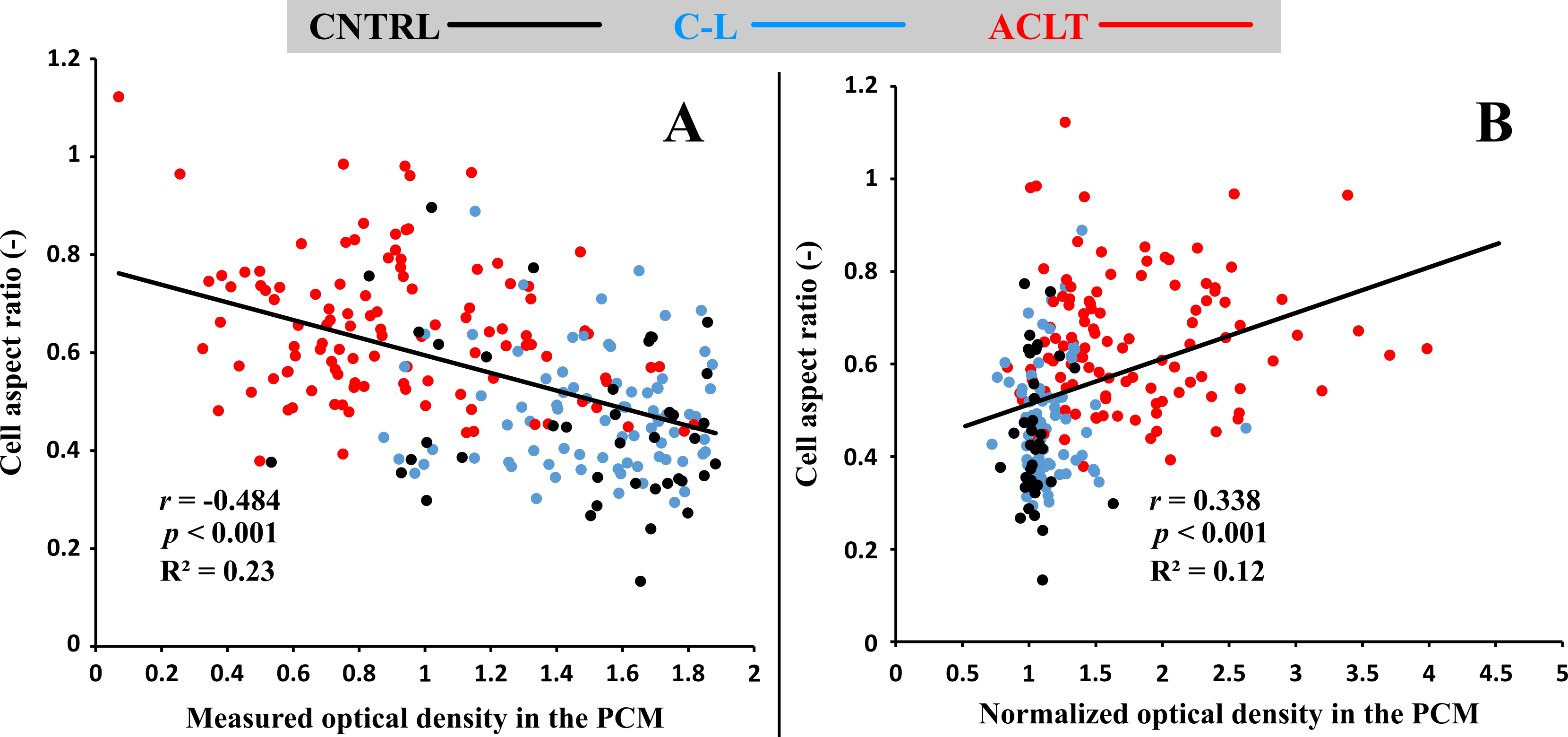

Supplement: S5 Fig — Correlation plot of the measured optical density and cell aspect ratio (r = -0.484, p < 0.001, R2 = 0.23) (A) and normalized optical density and cell aspect ratio (r = 0.338, p < 0.001, R2 = 0.12) (B) in the lateral femoral condyle cartilage. Red, blue and black dots represents the data of operated, contralateral and control groups, respectively. ACLT, Anterior Cruciate Ligament Transection; C-L, Contralateral; CNTRL, Control. (TIF) [file pone.0196203.s005.tif]
