# Supplementary material for: Site-specific glycosaminoglycan content is better maintained in the pericellular matrix than the extracellular matrix in early post-traumatic osteoarthritis
Source: PLoS One. 2018 Apr 25;13(4):e0196203. doi: 10.1371/journal.pone.0196203 (PMC5919041; doi:10.1371/journal.pone.0196203)
Supplement: S1 Table — * p<0.05, compared to the control group, ** p<0.05, comparison between the operated and contralateral groups. ACLT, Anterior Cruciate Ligament Transection; C-L, Contralateral; CNTRL, Control. (DOCX) [file pone.0196203.s006.docx]

**S1 Table: Mean values (95% CI) of the cell height, width and aspect ratio (height divided by width) in the superficial zone of the femoral groove, patella and lateral and medial femoral condyle and tibial plateau.**

| *Site* | *Cell* |  |  |  |  |  |  |
| --- | --- | --- | --- | --- | --- | --- | --- |
| *Femoral groove* | | ACLT | (95% CI) | C-L | (95% CI) | CNTRL | (95% CI) |
|  | *Height* | 7.67 | (7.12-8.23) | 7.38 | (6.74-8.03) | 8.55 | (7.65-9.44) |
|  | *Width* | 14.22 | (13.52-14.90) | 13.14 | (12.33-13.94) | 13.52 | (12.41-14.63) |
|  | *Aspect ratio* | 0.55 | (0.51-0.58) | 0.57 | (0.52-0.61) | 0.66 | (0.60-0.72) |
| *Lateral femoral condyle* | | ACLT | (95% CI) | C-L | (95% CI) | CNTRL | (95% CI) |
|  | *Height* | 7.074 | (6.58-7.56)* | 6.23 | (5.59-6.87) | 5.55 | (4.74-6.35) |
|  | *Width* | 11.05 | (10.53-11.56)*,** | 12.91 | (12.24-13.60) | 12.75 | (11.90-13.60) |
|  | *Aspect ratio* | 0.64 | (0.59-0.70)*,** | 0.49 | (0.42-0.57) | 0.44 | (0.35-0.54) |
| *Medial femoral condyle* | | ACLT | (95% CI) | C-L | (95% CI) | CNTRL | (95% CI) |
|  | *Height* | 6.67 | (5.97-7.38) | 5.94 | (4.91-6.97) | 6.09 | (4.94-7.23) |
|  | *Width* | 11.51 | (10.95-12.07) | 11.66 | (10.85-12.48) | 12.67 | (11.74-13.60) |
|  | *Aspect ratio* | 0.58 | (0.52-0.64) | 0.52 | (0.44-0.60) | 0.48 | (0.39-0.58) |
| *Lateral tibial plateau* | | ACLT | (95% CI) | C-L | (95% CI) | CNTRL | (95% CI) |
|  | *Height* | 7.32 | (6.70-7.93) | 7.91 | (7.14-8.68) | 7.72 | (6.86-8.57) |
|  | *Width* | 10.26 | (9.70-10.82) | 11.14 | (10.43-11.85) | 11.019 | (10.26-11.78) |
|  | *Aspect ratio* | 0.7 | (0.65-0.79) | 0.72 | (0.63-0.80) | 0.7 | (0.61-0.80) |
| *Medial tibial plateau* | | ACLT | (95% CI) | C-L | (95% CI) | CNTRL | (95% CI) |
|  | *Height* | 7.88 | (7.43-8.32) | 7.61 | (7.22-8.00) | 8.46 | (7.87-9.04) |
|  | *Width* | 10.18 | (9.67-10.70)* | 9.81 | (9.37-10.25)* | 11.3 | (10.70-11.90) |
|  | *Aspect ratio* | 0.8 | (0.74-0.85) | 0.8 | (0.76-0.84) | 0.75 | (0.70-0.81) |
| *Patella* | | ACLT | (95% CI) | C-L | (95% CI) | CNTRL | (95% CI) |
|  | *Height* | 8.34 | (7.67-9.00) | 9.19 | (8.11-10.26) | 9.12 | (7.89-10.34) |
|  | *Width* | 11.12 | (10.73-11.51) | 10.96 | (10.27-11.65) | 12.02 | (11.31-12.74) |
|  | *Aspect ratio* | 1.41 | (1.29-1.51) | 1.24 | (1.04-1.45) | 1.4 | (1.17-1.63) |
| ACLT, Anterior Cruciate Ligament Transection; C-L, Contralateral; CNTRL, Control; CI, Confidence Interval.  *p-*values were calculated using Bonferroni corrected pairwise comparison.  * *p*<0.05, compared to the CNTRL group.  ** *p*<0.05, comparison between the ACLT and C-L groups. | | | | | | | |
