# Supplementary material for: Site-specific glycosaminoglycan content is better maintained in the pericellular matrix than the extracellular matrix in early post-traumatic osteoarthritis
Source: PLoS One. 2018 Apr 25;13(4):e0196203. doi: 10.1371/journal.pone.0196203 (PMC5919041; doi:10.1371/journal.pone.0196203)
Supplement: S2 Table — * p<0.05, compared to the control group, ** p<0.05, comparison between the operated and contralateral groups. ACLT, Anterior Cruciate Ligament Transection; C-L, Contralateral; CNTRL, Control. (DOCX) [file pone.0196203.s007.docx]

| *Site* | *Cell* |  |  |  |  |  |  |
| --- | --- | --- | --- | --- | --- | --- | --- |
| *Femoral groove* | | ACLT | (95% CI) | C-L | (95% CI) | CNTRL | (95% CI) |
|  | *Height* | 10.94 | (10.07-11.82) | 11.7 | (10.82-12.58) | 11.07 | (9.65-12.49) |
|  | *Width* | 12.78 | (11.78-13.63) | 12.92 | (12.04-13.80) | 13.32 | (11.96-14.68) |
|  | *Aspect ratio* | 0.89 | (0.81-0.96) | 0.94 | (0.86-1.01) | 0.84 | (0.72-0.96) |
| *Lateral femoral condyle* |  | ACLT | (95% CI) | C-L | (95% CI) | CNTRL | (95% CI) |
|  | *Height* | 10.87 | (10.28-11.50)*,** | 12.3 | (11.63-12.97) | 12.44 | (11.42-13.45) |
|  | *Width* | 11.67 | (11.18-12.17) | 13.87 | (13.30-14.45) | 13.15 | (12.28-14.02) |
|  | *Aspect ratio* | 0.94 | (0.89-1.00) | 0.9 | (0.84-0.96) | 0.96 | (0.87-1.04) |
| *Medial femoral condyle* |  | ACLT | (95% CI) | C-L | (95% CI) | CNTRL | (95% CI) |
|  | *Height* | 10.86 | (10.26-11.46) | 11.56 | (10.61-12.52) | 12.37 | (11.39-13.35) |
|  | *Width* | 12.08 | (11.38-12.78) | 12.77 | (11.64-13.90) | 12.99 | (11.83-14.15) |
|  | *Aspect ratio* | 0.91 | (0.87-0.95) | 0.91 | (0.84-0.98) | 0.97 | (0.90-1.04) |
| *Lateral tibial plateau* |  | ACLT | (95% CI) | C-L | (95% CI) | CNTRL | (95% CI) |
|  | *Height* | 11.12 | (10.46-11.77)* | 11.7 | (10.77-12.63)* | 14.28 | (13.69-14.88) |
|  | *Width* | 11.62 | (10.74-12.49) | 12.53 | (11.35-13.72) | 13.47 | (12.18-14.76) |
|  | *Aspect ratio* | 0.98 | (0.90-1.06) | 0.95 | (0.84-1.05) | 1.05 | (0.94-1.16) |
| *Medial tibial plateau* |  | ACLT | (95% CI) | C-L | (95% CI) | CNTRL | (95% CI) |
|  | *Height* | 10.59 | (9.70-11.47)* | 9.95 | (9.06-10.85)* | 14.83 | (13.71-15.95) |
|  | *Width* | 10.94 | (10.07-11.80)* | 10.89 | (10.04-11.73)* | 14.01 | (12.80-15.27) |
|  | *Aspect ratio* | 0.98 | (0.92-1.04) | 0.94 | (0.88-1.00) | 1.06 | (0.99-1.14) |
| *Patella* |  | ACLT | (95% CI) | C-L | (95% CI) | CNTRL | (95% CI) |
|  | *Height* | 11.53 | (10.48-12.57)* | 12.6 | (11.23-13.97) | 14.4 | (12.47-16.32) |
|  | *Width* | 11.9 | (10.94-12.85) | 13.06 | (10.81-14.30) | 13.61 | (11.84-15.37) |
|  | *Aspect ratio* | 1.06 | (1.02-1.10) | 1.06 | (1.00-1.12) | 0.98 | (0.90-1.05) |
| ACLT, Anterior Cruciate Ligament Transection; C-L, Contralateral; CNTRL, Control; CI, Confidence Interval.  *p-*values were calculated using Bonferroni corrected pairwise comparison.  * *p*<0.05, compared to the CNTRL group.  ** *p*<0.05, comparison between the ACLT and C-L groups. | | | | | | | |

**S2 Table: Mean values (95% CI) of the cell height, width and aspect ratio (height divided by width) in the middle zone of the femoral groove, patella and lateral and medial femoral condyle and tibial plateau.**
