# Supplementary material for: Site-specific glycosaminoglycan content is better maintained in the pericellular matrix than the extracellular matrix in early post-traumatic osteoarthritis
Source: PLoS One. 2018 Apr 25;13(4):e0196203. doi: 10.1371/journal.pone.0196203 (PMC5919041; doi:10.1371/journal.pone.0196203)
Supplement: S3 Table — * p<0.05, compared to the control group, ** p<0.05, comparison between the operated and contralateral groups. ACLT, Anterior Cruciate Ligament Transection; C-L, Contralateral; CNTRL, Control. (DOCX) [file pone.0196203.s008.docx]

| *Site* | *Cell* |  |  |  |  |  |  |
| --- | --- | --- | --- | --- | --- | --- | --- |
| *Femoral groove* | | ACLT | (95% CI) | C-L | (95% CI) | CNTRL | (95% CI) |
|  | *Height* | 15.11 | (13.19-17.03) | 14.81 | (13.37-16.26) | 14.42 | (12.43-16.42) |
|  | *Width* | 12.27 | (10.79-13.76)* | 11.91 | (10.81-13.00)* | 15.19 | (13.72-16.66) |
|  | *Aspect ratio* | 1.25 | (1.05-1.45) | 1.31 | (0.16-1.45) | 1.03 | (0.83-1.23) |
| *Lateral femoral condyle* |  | ACLT | (95% CI) | C-L | (95% CI) | CNTRL | (95% CI) |
|  | *Height* | 15.37 | (14.24-16.51) | 15.81 | (14.60-17.03) | 17.52 | (15.71-19.34) |
|  | *Width* | 11.03 | (10.44-11.61) | 10.62 | (9.93-11.31) | 11.46 | (10.40-12.53) |
|  | *Aspect ratio* | 1.41 | (1.31-1.51) | 1.49 | (1.38-1.61) | 1.56 | (1.39-1.73) |
| *Medial femoral condyle* |  | ACLT | (95% CI) | C-L | (95% CI) | CNTRL | (95% CI) |
|  | *Height* | 14.73 | (13.37-16.08) | 17.38 | (15.57-19.20) | 17.66 | (15.66-19.66) |
|  | *Width* | 10.44 | (9.73-11.15) | 11.34 | (10.39-12.29) | 11.04 | 10.01-12.07) |
|  | *Aspect ratio* | 1.43 | (1.34-1.52) | 1.54 | (1.42-1.66) | 1.54 | (1.41-1.66) |
| *Lateral tibial plateau* |  | ACLT | (95% CI) | C-L | (95% CI) | CNTRL | (95% CI) |
|  | *Height* | 18.06 | (16.34-19.78) | 20.32 | (18.24-22.40) | 19.89 | (17.41-22.37) |
|  | *Width* | 12.02 | (10.83-13.21) | 12.84 | (11.39-14.29) | 14.34 | (12.39-16.29) |
|  | *Aspect ratio* | 1.56 | (1.40-1.71) | 1.68 | (1.50-1.86) | 1.43 | (1.16-1.70) |
| *Medial tibial plateau* |  | ACLT | (95% CI) | C-L | (95% CI) | CNTRL | (95% CI) |
|  | *Height* | 20.98 | (18.96-23.01) | 21.01 | (18.83-23.19) | 21.9 | (19.40-24.40) |
|  | *Width* | 13.6 | (12.70-14.51)** | 11.71 | (10.82-12.61)* | 14.67 | (14.07-15.27) |
|  | *Aspect ratio* | 1.59 | (1.43-1.76) | 1.84 | (1.66-2.01) | 1.53 | (1.30-1.75) |
| *Patella* |  | ACLT | (95% CI) | C-L | (95% CI) | CNTRL | (95% CI) |
|  | *Height* | 21.26 | (20.19-22.34) | 22.28 | (20.77-23.78) | 20.78 | (18.98-22.57) |
|  | *Width* | 17.19 | (16.07-18.32) | 16.97 | (15.46-18.49) | 17.1 | (15.18-19.03) |
|  | *Aspect ratio* | 0.83 | (0.79-0.86) | 0.81 | (0.74-0.87) | 0.86 | (0.80-0.93) |
| ACLT, Anterior Cruciate Ligament Transection; C-L, Contralateral; CNTRL, Control; CI, Confidence Interval.  *p-*values were calculated using Bonferroni corrected pairwise comparison.  * *p*<0.05, compared to the CNTRL group.  ** *p*<0.05, comparison between the ACLT and C-L groups. | | | | | | | |

**Table 3: Mean values (95% CI) of the cell height, width and aspect ratio (height divided by width) in the deep zone of the femoral groove, patella and lateral and medial femoral condyle and tibial plateau.**
